# Molecular Characterization of a New *Babesia bovis* Thrombospondin-Related Anonymous Protein (BbTRAP2)

**DOI:** 10.1371/journal.pone.0083305

**Published:** 2013-12-13

**Authors:** Mohamad Alaa Terkawi, Jadsada Ratthanophart, Akram Salama, Mahmoud AbouLaila, Masahito Asada, Akio Ueno, Hend Alhasan, Azirwan Guswanto, Tatsunori Masatani, Naoaki Yokoyama, Yoshifumi Nishikawa, Xuenan Xuan, Ikuo Igarashi

**Affiliations:** 1 National Research Center for Protozoan Diseases, Obihiro University of Agriculture and Veterinary Medicine, Obihiro, Hokkaido, Japan; 2 Department of Parasitology, Faculty of Veterinary Medicine, Sadat City University, Minoufiya, Egypt; 3 Department of Animal Medicine and Infectious Diseases, Faculty of Veterinary Medicine, Sadat City University, Minoufiya, Egypt; Federal University of São Paulo, Brazil

## Abstract

A gene encoding a *Babesia bovis* protein that shares significant degree of similarity to other apicomplexan thrombospondin-related anonymous proteins (TRAPs) was found in the genomic database and designated as *BbTRAP2*. Recombinant protein containing a conserved region of BbTRAP2 was produced in *E. coli*. A high antigenicity of recombinant BbTRAP2 (rBbTRAP2) was observed with field *B. bovis*-infected bovine sera collected from geographically different regions of the world. Moreover, antiserum against rBbTRAP2 specifically reacted with the authentic protein by Western blot analysis and an indirect fluorescent antibody test. Three bands corresponding to 104-, 76-, and 44-kDa proteins were identified in the parasite lysates and two bands of 76- and 44-kDa proteins were detected in the supernatant of cultivated parasites, indicating that BbTRAP2 was proteolytically processed and shed into the culture. Apical and surface localizations of BbTRAP2 were observed in the intracellular and extracellular parasites, respectively, by confocal laser microscopic examination. Moreover, native BbTRAP2 was precipitated by bovine erythrocytes, suggesting its role in the attachment to erythrocytes. Furthermore, the specific antibody to rBbTRAP2 inhibited the growth of *B. bovis* in a concentration-dependent manner. Consistently, pre-incubation of the free merozoites with the antibody to rBbTRAP2 resulted in an inhibition of the parasite invasion into host erythrocytes. Interestingly, the antibody to rBbTRAP2 was the most inhibitive for the parasite’s growth as compared to those of a set of antisera produced against different recombinant proteins, including merozoite surface antigen 2c (BbMSA-2c), rhoptry-associated protein 1 C-terminal (BbRAP-1CT), and spherical body protein 1 (BbSBP-1). These results suggest that BbTRAP2 might be a potential candidate for development of a subunit vaccine against *B. bovis* infection.

## Introduction

*Babesia bovis* is tick-borne haemoprotozoan parasite of cattle that causes significant economic losses in dairy and beef industries. Typically, the infection is characterized by haemolytic anemia, hyperpyrexia, hemoglobinuria, lethargy, inappetence, and sometimes hydrophobia [1]. Fatal disturbances may occur when the infected erythrocytes (iRBCs) sequestrate in the microcapillaries of kidneys, lungs, and the brain, resulting in organ failure and systemic shock [1–3]. Despite the fact that chemotherapy is still the mainstay for treatment and control, the high prevalence of infection worldwide and the emergence of drug resistance [3] have spurred an interest in developing more effective measures that can counter the spread of infection and reduce its significant impact of the infection on livestock industry. Attenuated vaccines offer a reasonably long-lasting protection; however, the possible spread of silent pathogens such as leukemia virus, difficulties in standardizing the vaccine dose, and the risk of reversion of virulence have restricted the use of this type of vaccine in many regions of the world [4,5]. Vaccines based on killed parasites and soluble parasite antigens derived from different *Babesia* species have shown partial protection characterized by reduction of the manifestations of clinical disease in animals [6,7]. Recently, the efforts of vaccine development have shifted toward the use of antigenically defined immunogens, particularly the molecules interacting or disrupting the process of parasite invasion into host RBCs [8]. 

The invasion process is an essential step in the life cycle of apicomplexan parasites and is dependent on the interaction between the parasite- and host-surface molecules [9,10]. In *Plasmodium* spp, the extracellular merozoites are considered to initially establish a reversible attachment with the RBCs via glycosyl phosphatidylinositol anchor (GPI) of merozoite surface proteins (MSPs). The merozoite then re-orientates bringing the anterior apical pole into contact with the plasma membrane of RBCs [9], and at this point, micronemes and rhoptries release higher-affinity transmembrane adhesins leading to irreversible attachment with the RBC surface and the formation of tight junction [10,11]. The parasites then actively invade host cells through a moving junction mediated by apical membrane antigen 1 (AMA1) and rhoptry neck protein (RON) and in a process driven by an actomyosin motor [11,12]. More recent study has shown that the AMA1-RON2 interaction does not have an essential role at tight junction of apicomplexan parasites but they may act separately during the invasion [13]. The model of *Babesia* invasion is still speculated and relied on the data obtained from *Plasmodium* spp. [9]. Although these molecules were all identified in *Babesia* parasites, the precise mechanism of invasion into RBCs, including such as tight junction, remains obscure and needs further investigation. Nonetheless, secreted proteins from microneme are believed to play a key role in parasite invasion and have been received the major research focus in vaccine development [9–12]. 

Members of the thrombospondin-related anonymous protein (TRAP) family are micronemal proteins that are present in all apicomplexan parasites with conserved structures, consisting of a hydrophobic short N-terminal sequence, a von Willebrand factor A (vWFA) region, thrombospondin type 1 (TSP-1) domains, and a hydrophobic transmembrane sequence followed by a short cytoplasmic tail [14]. They are proposed to function as adhesive molecules involved in the cell–matrix interactions via the vWFA and TSP1 domains [14]. Interestingly, homologues of *TRAP* were identified in all of the invasive stages of *Plasmodium* spp, including sporozoite (*TRAP*), merozoite (*MTRAP*), and ookinete (*WARP*), as essential genes for survival of the parasite [15–17]. Moreover, the TRAP orthologues have been identified in several other protozoa, including *Toxoplasma gondii*, *Neospora caninum*, *Cryptosporidium* spp, *Eimeria* spp, and *Babesia* spp [14–17]. The homologues of *TRAP* were identified in *B. bovis* and *B. gibsoni* to be expressed during the asexual blood stages, and the antibodies to their recombinant proteins significantly inhibited parasite invasion to host RBCs [18,19]. Therefore, identification and characterization of the genes encoding TRAPs in *Babesia* spp are beneficial in the discovery and development of molecular babesial vaccine. 

Bioinformatics analyses against the genomic sequence database of *B. bovis* (T2Bo strain) deposited recently in the GenBank [20] revealed the presence of at least four genes encoding *B. bovis TRAPs*. Among them, BbTRAP2 showed the highest gene expression in erythrocytic stage and identities with *B. gibsoni* P18 (BgTRAP2) known as potential vaccine candidate against canine babesiosis [19]. Therefore in the present study, we characterized a TRAP2 as a new vaccine candidate against *B. bovis* infection.

## Materials and Methods

### Ethics Statement

All animal experiments described in this article were conducted in accordance with the Guiding Principles for the Care and Use of Research Animals Promulgated by Obihiro University of Agriculture and Veterinary Medicine, Japan. The protocol was approved by the Committee on the Ethics of Animal Experiments of the Obihiro University of Agriculture and Veterinary Medicine (Permit number 23–26).

### Parasite

A *B. bovis* Texas strain (T2Bo strain) was continuously cultivated in bovine RBCs, using a microaerophilous stationary-phase culturing system, and the iRBCs were harvested at the peak-parasitemia and then stored at -80 °C for further use [21]. Medium M199 (Sigma-Aldrich, Tokyo, Japan) supplemented with 40 % normal bovine serum, 60 U/ml of penicillin G, 60 µg/ml of streptomycin, and 0.15 µg/ml of amphotericin B (Sigma Aldrich) was used for maintaining the parasites culture in fresh bovine RBCs. 

### Identification of *B. bovis* TRAP paralogues

The genomic database of *B. bovis* T2Bo (http://www.genome.jp/kegg/) was screened to identify the TRAP paralogues using a pBLAST (http://blast.ncbi.nlm.nih.gov). The genes, which have significant degree of similarity to previously reported *BbTRAP* [18], were analyzed by SMART (http://smart.embl-heidelberg.de/) for the presence of TSP1 and vWFA domains and by a TMHMM server (http://www.cbs.dtu.dk/services/TMHMM-2.0/) for the presence of a transmembrane domain. 

### Reverse transcription-polymerase chain reaction

A total RNA of *B. bovis*-iRBCs was extracted using a TRI reagent (Sigma) and then subjected to a one-step RT-PCR kit with the specific primers of BbTRAPs (Table S1) according to the manufacturer’s instructions (Takara, Tokyo, Japan), after the treatment of recombinant DNase (Takara). The amplified cDNAs were used as templates for sequencing using an automated sequencer (ABI PRISM 3100 Genetic Analyzer, Foster City, CA, USA), and the obtained sequences were finally analyzed by the BLSAT as described above. For quantifying the gene expression, the RT-PCR bands were analyzed by ImageJ software (http://rsbweb.nih.gov/ij/index.html).

### Production of recombinant proteins and preparation of polyclonal antibodies

A cDNA encoding the target region of BbTRAP2 (Ser^341^-Glu^920^) was amplified by using a set of primers: 5’-ATGAATTCCCTCGAATCGTGGTAATTTTAGCAACG-3’ and 5’-AAGCGGCCGCTTATTCCACCTTCGGGATTAC-3’ with the underlined restriction sites of *Eco*RI and *Not*I, respectively. The resulting PCR product was subcloned into a pGEX6p2 vector (Amersham Pharmacia Biotech, Madison, CA, USA) using the suitable restriction enzyme sites and then expressed as a glutathione S-transferase (GST)-fusion in an *E. coli* DH-5α strain (Amersham Pharmacia Biotech). The recombinant protein was purified from the soluble fraction of *E. coli* lysate using a Glutathione-Sepharose 4B bead (Amersham Pharmacia Biotech). Thereafter, antisera against rBbTRAP2 and control GST protein were prepared in six-week-old ICR mice (n=5) and Japanese white rabbits (n=2) (CLEA, Tokyo, Japan) according to the standard protocols [21]. Briefly, the mice were intraperitoneally (i.p.) immunized with 100 µg of the recombinant protein emulsified in Freund’s complete adjuvant (Sigma). Three boosters were given i.p. using same protein emulsified in Freund’s incomplete adjuvant (Sigma) at 14-day intervals. Following the same procedure, the rabbits were subcutaneously immunized with 1 mg of purified recombinant protein and then boosted three times with the same protein at 14-day intervals. Total immunoglobin Gs (IgGs) were then purified from the rabbit sera through a Protein A chromatography column according to the manufacturer’s instructions (Bio-Rad Laboratories, Hercules, CA, USA). Specific antibodies to BbMSA-2c, BbRAP-1CT, and BbSBP-1 were prepared in rabbits following same protocol, and all prepared sera had >12,800 titration against parasites as determined by an immunofluorescence test [21]. 

### SDS-PAGE and Western blot analyses

The expressed recombinant proteins were verified by sodium dodecyl sulfate-polyacrylamide gel electrophoresis (SDS-PAGE) with subsequent Coomassie blue staining. Furthermore, *B. bovis* authentic BbTRAP2 was identified using Western blot analysis [21]. Briefly, *B. bovis*-iRBCs obtained from the *in vitro* culture were washed with cold phosphate-buffered saline (PBS) and then lysed in 25-fold distilled water (DW) (v:v). The pellet was washed four times with cold PBS, disrupted three times by a freeze-thaw cycle in liquid nitrogen, and then sonicated in ice slurry. The blotted membrane was blocked with 0.05% Tween 20 in PBS (PBS-T) plus 5% skimmed milk and then probed with anti-rBbTRAP2 rabbit or mouse serum (1:500). A secondary antibody (1:4,000) of horseradish peroxidase (HRP)-conjugated anti-rabbit or -mouse IgG (Bethyl Laboratories, Montgomery, TX, USA) was used to identify the bound proteins on the blots. Finally, the positive bands were visualized using a solution containing 3-diaminobenzidine tetrahydrochloride DAB and H_2_O_2_ (Dojindo, Tokyo, Japan). 

### Enzyme-linked immunosorbent assay (ELISA)

An ELISA was performed to examine the antigenicity of rBbTRAP2 against a set of bovine sera collected from experimentally and naturally *B. bovis*-infected cattle, which were stocked at our laboratory [21]. Briefly, 96-well microtiter plates (Nunc, Roskilde, Denmark) were coated overnight at 4°C with 50 µl of rBbTRAP2 at a concentration of 2 µg/ml in a coating buffer (50 mM carbonate-bicarbonate buffer, pH 9.6). The antigen-coated wells were blocked with 3% skimmed milk in PBS for 1 h at 37°C and then incubated with 50 µl of the serum samples diluted 1:100 in the blocking solution for 1 h at 37°C. After appropriate washing with PBS-T, the plates were incubated with a secondary antibody of HRP-conjugated sheep anti-bovine IgG (Bethyl) in the blocking solution (1:4,000) for 1 h at 37°C. Thereafter, the plates were washed six times with PBS-T, and 100 µl of a substrate solution [0.1 M citric acid, 0.2 M sodium phosphate, 0.3 mg/ml 2,2’-azide-bis (3-ethylbenzthiazoline-6-sulfonic acid) (Sigma), and 0.01% of a 30% hydrogen peroxide solution in DW (v:v)] was added to each well. The absorbance at 415 nm was measured after 1 h of incubation at room temperature (RT) using an MTP-500 microplate reader (Corona Electric, Tokyo, Japan). 

### Confocal laser microscopic examination

An indirect fluorescent antibody test (IFAT) was performed to determine the cellular localization of BbTRAP2 [22]. Briefly, thin blood smears of *B. bovis*-iRBCs were fixed in a solution of 95% methanol and 5% acetone (v:v) at -20°C for 30 min and then probed with anti-rBbTRAP2 immune sera diluted in 3% fetal bovine serum in PBS (1:200) for 1 h at 37°C in a moist chamber. A secondary antibody of Alexa-Fluor® 488-conjugated goat anti-rabbit or -mouse IgG (Molecular Probes, Invitrogen, Carlsbad, CA, USA) diluted in the same buffer (1:400) was applied on the smears and then incubated for 30 min at 37°C. For nuclear staining, the smears were then incubated with a Hoechst solution (Dojindo) in PBS (1:100) for 10 min at RT. After washing with PBS-T, the smears were mounted using a fluorescent mounting medium (Dako, Carpinteria, CA, USA) and then covered with a glass coverslip. For staining the live parasites, the Hoechst solution was added in advance to the medium of cultivated iRBCs (1:100) and then incubated for 15 min at 37°C. Thereafter, the supernatant was replaced with a GIT medium (Wako, Osaka, Japan) containing the anti-rBbTRAP2 rabbit serum (1:100), and the culture was then incubated for 1 h at 37°C. After being washed four times with the medium, Alexa-Fluor® 488-conjugated goat anti-rabbit IgGs were added to the medium (1:400), and the culture was then incubated for 30 min at 37°C. After washing twice each with the medium and then with PBS, thin smears were made and fixed with methanol at -20°C for 30 min. Imaging was performed using a confocal laser scanning microscope (TCS NT, Leica, Heidelberg, Germany). For the co-localization study, anti-rBbAMA1 mouse serum [23] was used as a specific marker for microneme of the parasites. 

### Immunoprecipitation assay

The iRBCs were washed with cold PBS and then lysed in 25-fold DW. The pellet was washed four times with cold PBS, disrupted three times by a freeze-thaw cycle in liquid nitrogen, and then stored as the lysate of parasites at -30°C for an immunoprecipitation assay. The supernatant of iRBCs was also harvested at the peak-parasitemia, centrifuged at 13,000 × *g* for 60 min, and then stored at -30°C for the assay. Purified rabbit anti-rBbTRAP2 IgG (0.2-0.5 mg) was covalently immobilized on 20μl pierce protein A/G plus agarose (Crosslink Immunoprecipitation Kit) according to the manufacturer’s instructions (Thermo Fisher Scientific, Inc., Rockford, IL, USA). A soluble fraction of the parasite lysate (0.25 mg) or supernatant of the culture (100μl) was incubated with the antibody-bound beads for 2 h at RT. The precipitated proteins were eluted, resolved by SDS-PAGE, and then detected by Western blot analyses using the anti-rBbTRAP mouse serum, as described above.

### Bovine RBC binding assay

RBC binding assay was performed on the whole cells and membrane ghosts as previously described [24], with some modifications. Briefly, 100 µl of bovine RBCs with a 50% hematocrit was washed three times in an incomplete RPMI 1640 medium (Sigma) and then resuspended in 500 μl of the RPMI 1640 medium coupled with a soluble fraction of the parasite lysate in 1 ml of PBS (0.5 mg). The membrane ghosts were prepared from 100 µl of bovine RBCs lysed in 20-folds buffer containing 5mM Tris (pH= 7.4), 1mM EDTA and 7mM sodium chloride, washed three times with PBS and one time in RPMI 1640 medium (Sigma), and then resuspended in 500 μl of the RPMI 1640 medium coupled with a soluble fraction of the parasite lysate in 1 ml of PBS (0.5 mg). The mixture was incubated at 37°C for 1 h with constant shaking and then centrifuged at 13,000 × *g* for 30 sec. Thereafter, the RBCs were washed five times with 10-fold RPMI 1640 medium and then washed once with PBS. Bound proteins were eluted from the RBC pellet by the addition of 200 μl of 1 M NaCl and then analyzed by Western blotting using the anti-rBbTRAP2 rabbit serum, or anti-GST and anti-ribosomal phosphoprotein sera as negative control for validating the reaction [25]. 

### In vitro growth inhibition assay

The growth inhibition assay using cultured *B. bovis* was performed as described previously [26]. Briefly, bovine iRBCs at 8-10% parasitemia were diluted with fresh bovine RBCs to achieve 1% parasitemia and 10% packed cell volume (PCV). Medium GIT (Wako) was prepared with tested IgG to a final concentration of 0.25, 0.5, or 1 mg/ml, while control anti-GST IgG was used at a 1 mg/ml concentration. Free GIT medium without antibody was used as control. Two hundred microliters of the mixture containing 20 μl of 1% iRBCs was dispensed into 96-well plates (Nunc) and then incubated at 37°C in a humidified multigas water-jacketed incubator (90% N_2_, 5% CO_2_, 5% O_2_) for 4 days. Moreover, rabbit antibody to BbMSP-1c, BbRAP-1CT and BbSBP-1 was used as positive control for comparison [21,27,28]. Furthermore, we examined the growth inhibition in the presence of cytochalasin D (Sigma) and antibody as earlier described [29] with some modifications. Briefly, *B. bovis* culture having 2% iRBCs were grown in GIT medium containing 25 or 100 nM Cytochalasin D and 1mg purified anti-BbTRAP2 IgG for two days. Giemsa-stained smears were made every day, and the parasitemias were determined on the basis of approximately 1,000 RBCs. The experiments were carried out in triplicate for each IgG concentration in at least two separate trials.

### In vitro invasion inhibition assay

The *B. bovis* invasion inhibition assay was performed as described earlier [18,30], with some modifications. Briefly, *B. bovis*-iRBCs were harvested at the peak-parasitemia, washed once, and then resuspended in an equal volume of GIT medium. Merozoites of *B. bovis* were liberated by five intermittent high-voltage pulses (1.25 kV, 300 Ω, and 25 μF) with 10 sec in ice between the pulses in 4-mm cuvettes (BioRad) using a BioRad Gene Pulser with pulse controller and then resuspended in a GIT medium containing the purified anti-rBbTRAP2 IgG or anti-GST IgG of 1 mg/ml. Control cultures were prepared without an antibody. After incubation of merozoites for 1 h at 20°C with antibody, bovine RBCs were added to the final PCV of 10%. Thereafter, 200μl of the mixture containing approximately 1×10^6^ free merozoites was dispensed into 96-well plates (Nunc) and then incubated at 37°C in a humidified multigas water-jacketed incubator for 6 h, covering one complete life cycle of *B. bovis*. Giemsa-stained smears were prepared after 1 and 6 h, and the parasitemia (maximum 1% after 6 h) was counted on the basis of approximately 3,000 RBCs. The percentages of merozoite invasion into RBCs were considered as a relation of intracellular parasites of test cultures to those of the controls. Experiments were carried out in triplicate for each IgG in two separate trials.

### Statistical analyses

The significant differences (GraphPad Prism 5, GraphPad Software, Inc., San Diego, CA, USA) among the means of all variables were examined by a one-way analysis of variance followed by Tukey’s multiple comparison test for pairwise comparison of data from the multiple groups. Results were considered to be statistically significant when *P* < 0.05.

## Results

### Identification of genes encoding *B. bovis* BbTRAPs and targeting analyses of *BbTRAP2*

Analyses of the *B. bovis* genomic database (T2Bo strain) revealed the presence of four genes encoding *BbTRAPs* that were designated as *BbTRAP1–4* (GenBank accession numbers XM_001609738, XM_001609762, XM_001609736, and XM_001609760, respectively) based on the identities to a previously reported BbTRAP of Israel strain (clonal line C61411, GenBank accession number AY486102) [18]. Notably, all four genes existed as a single copy gene in chromosome 2. Bioinformatics analyses demonstrated that BbTRAPs had typical structures of TRAPs from other apicomplexan parasites, as determined by the presence of vWFA, TSP1 domains, and a transmembrane domain followed by a cytoplasmic C-terminal tail containing a conserved tryptophan residue (Figure S1). Next, to examine the transcriptional expression of these genes in the merozoite stage, RNA was extracted from iRBCs and then examined by RT-PCR analyses. The results indicated that *BbTR*AP2 (*BbP18*) expression in the asexual stage of *B. bovis* was relatively higher than the expression of other *BbTRAPs* (Figure S1). Therefore, the gene encoding BbTRAP2 was selected for further characterization in the present study. The gene encoding *BbTRAP2* contains an ORF of 3,081 bp encoding a polypeptide of 1,027 amino acid residues with a high identity (41%) to *B. gibsoni* TRAP (BgP18) and a modest identity (25–27%) to other *TRAPs* of apicomplexan parasites, including *Theileria* spp and *Plasmodium* spp. BbTRAP2 contains a predicted signal peptide at the cleavage site between 25 and 26 amino acids, vWFA domains extending over amino acid residues His^68^-Ile^267^ and Asp^476^-Asn^637^, TSP1 domains extending over amino acid residues Glu^279^-Gly^340^ and Leu^685^-Cys^746^, and a transmembrane domain at Val^965^-Ile^984^. 

### Immune reactivity of recombinant BbTRAP2 against a set of *B. bovis*-infected bovine sera

**Figure 1:**
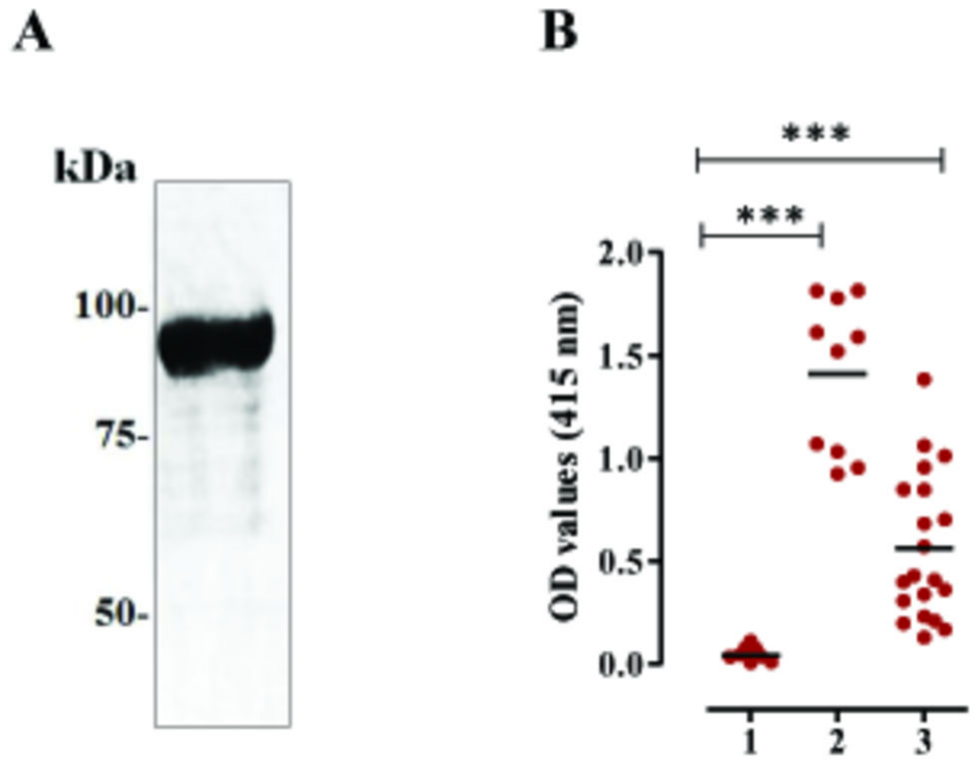
Recombinant BbTRAP2 expressed in *E. coli* and its antigenicity with bovine sera. **A**, SDS-PAGE of bacterial recombinant stained by Coomassie blue. **B**, The reactivity of rBbTRAP2 in ELISA with non-infected sera (lane 1), experimentally *B. bovis*-infected sera (n=10) (lane 2), and *B. bovis* sero-positive field sera (n=20) (lane 3). Infected bovine sera showed significantly higher OD values than non-infected sera. Asterisk indicates a significant difference (*P* < 0.0001). Horizontal lines in lane 1-3 indicate averages of OD values of tested sera.

To begin the molecular characterizations of BbTRAP2, a region encoding truncated BbTRAP2 (Ser^341^-Glu^920^) containing vWFA and TSP1 domains was expressed in *E. coli*. The targeted region showed a considerable conservation with other BbTRAPs, in which the identities ranged between 30 and 50% (Figure S2). The recombinant protein (rBbTRAP2) had 91-kDa molecular mass (Figure 1A), including a 26-kDa GST-tag. The antigenicity of rBbTRAP2 was evaluated with a set of experimentally and naturally *B. bovis*-infected sera. Notably, rBbTRAP2 demonstrated significantly higher OD values against both *B. bovis*-infected sera than those of non-infected sera (Figure 1B). The reactivities of rBbTRAP2 against field *B. bovis*-infected bovine sera collected from South Africa, Thailand, Mongolia, and Brazil indicated the elevation of a specific antibody to rBbTRAP2 in naturally infected cattle. These findings suggested that targeted region of BbTRAP2 is highly immunogenic and might be a potential vaccine candidate for *B. bovis*. 

### BbTRAP2 is functional in the merozoite stage of *B. bovis*

**Figure 2:**
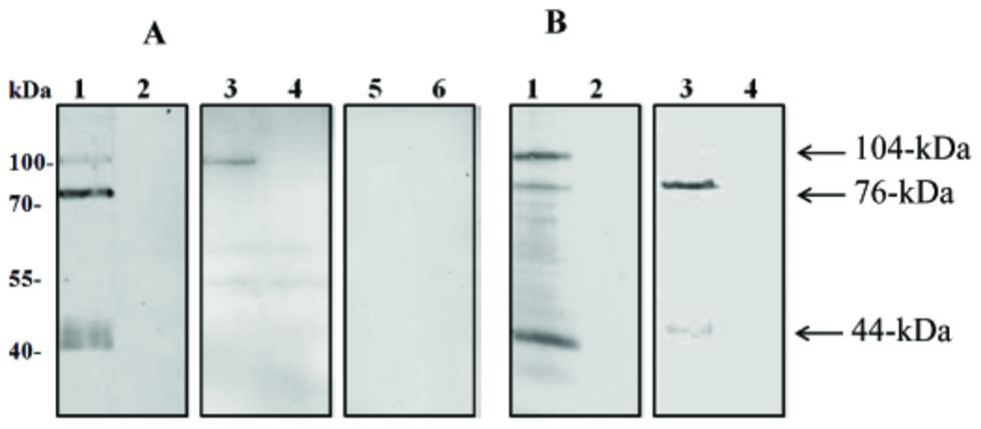
Western blot analyses for authentic BbTRAP2 with immune sera to BbTRAP2. **A**, whole lysates of *B. bovis* (lane 1), non-infected bovine RBCs (lane 2), insoluble fraction of *B. bovis* lysates (lane 3) and non-infected bovine RBCs (lane 4) probed with rabbit anti-rBbTRAP2 serum. Whole lysates of *B. bovis* (lane 5), insoluble fraction of *B. bovis* lysates and non-infected bovine RBCs (lane 6) blots were probed with pre-immune sera. **B**, Immunoprecipitated proteins by antibody to rBbTRAP2 from *B. bovis* lysate (lane 1), non-infected bovine RBCs (lane 2), supernatant of cultivated parasites (lane 3), and supernatant of non-infected bovine RBCs (lane 4).

**Figure 3:**
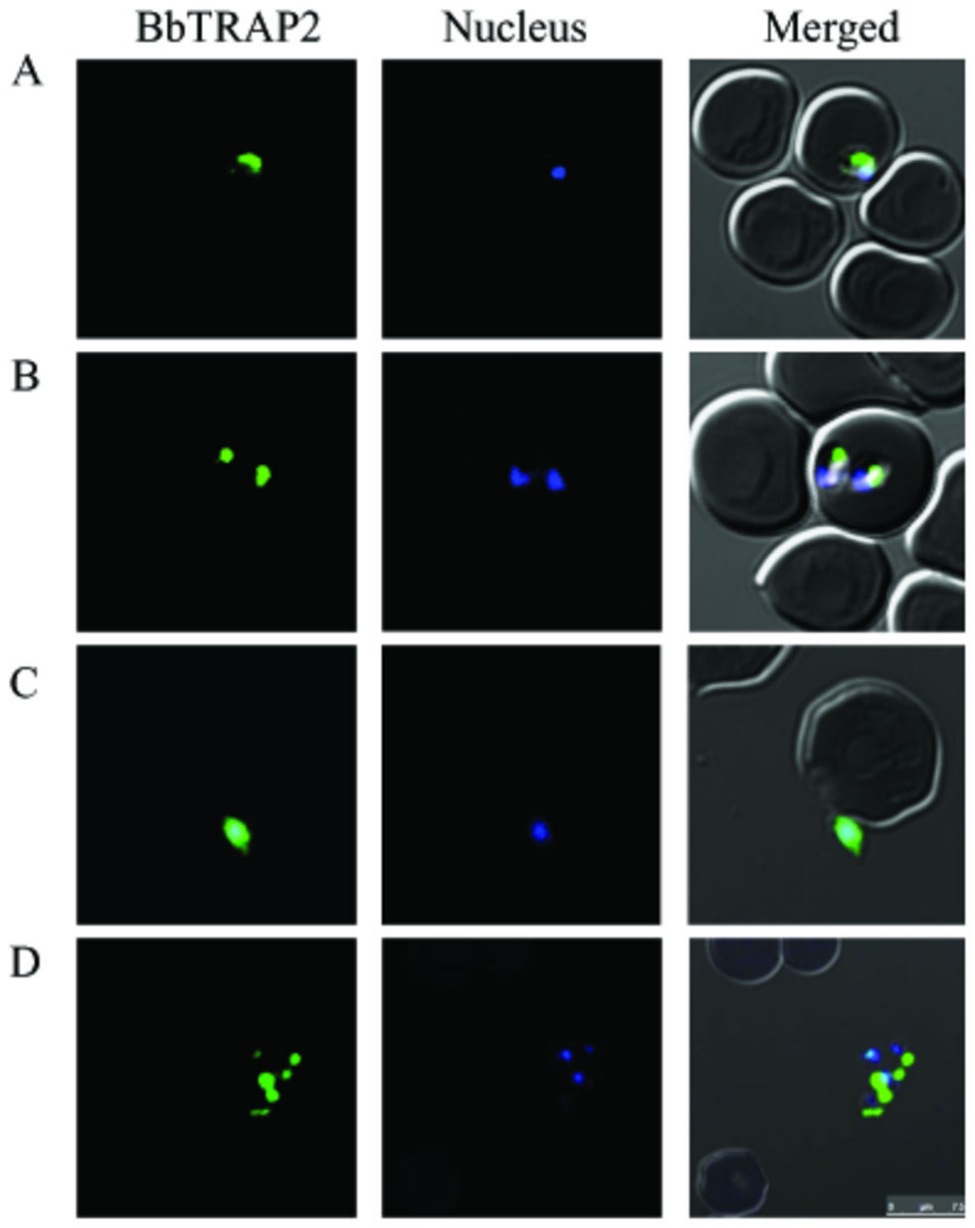
Cellular localization of BbTRAP2 in the erythrocytic stage. Confocal laser microscopic observation of BbTRAP2 in thin blood smears of *B. bovis*-parasitized RBCs stained with immune sera to BbTRAP2. Reactivity of the anti-rBbTRAP2 serum with intracellular parasites single form (A), sequentially dividing forms (B), and extracellular parasites (C, D). Pre-immune and anti-GST sera were used as negative control sera for validation of the test (data not shown). Scale bar: 7.5µm.

**Figure 4:**
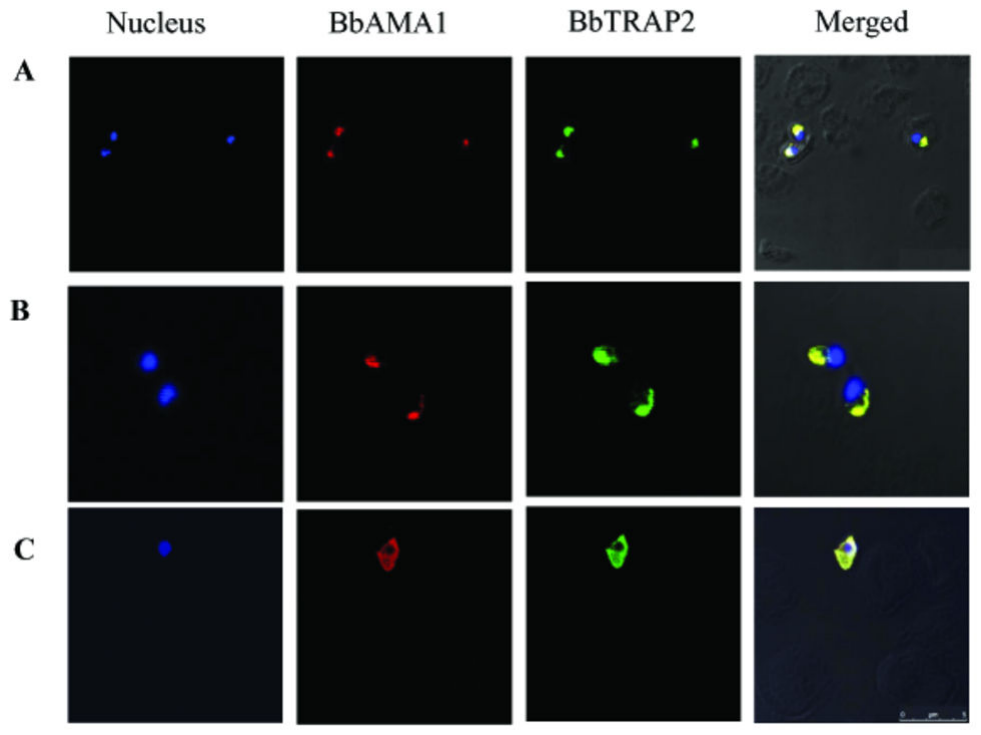
Co-localization study of BbTRAP2 and BbAMA1 by confocal laser microscopy. The reactivity of immune sera with intracellular (A, B) parasites and extracellular (C). Anti-BbAMA1 mouse serum (red), anti-BbTRAP2 (green), nuclear staining (blue). Scale bar: 5µm.

**Figure 5:**
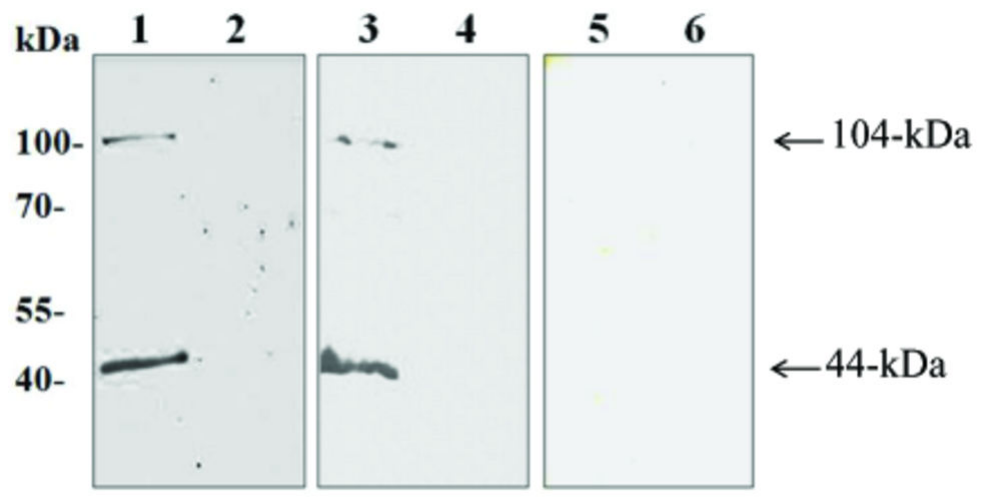
Western blot analyses for native BbTRAP2 precipitated by bovine RBCs. Lysates of *B. bovis* (lanes 1, 3, 5, 6) and bovine RBCs (lanes 2, 4) were incubated with intact bovine RBCs (lanes 1, 2, 5) and membrane ghost RBC (lanes 3, 4, 6), and precipitated proteins were detected by a specific immune rBbTRAP2 (lanes 1-4) or rP0 sera as negative control (lanes 5, 6).

To study the functional roles of BbTRAP2 in the erythrocytic stage of *B. bovis*, anti-rBbTRAP2 immune sera were produced in rabbits and mice. Rabbit immune serum was first used to identify the native proteins in the *B. bovis* lysate by Western blot analysis 3 bands corresponding to 104-, 76-, and 44-kDa native BbTRAP2 were detected in the *B. bovis* lysate but not in non-infected bovine RBCs (Figure 2A, lanes 1, 2). On the contrary, single band corresponding to 104-kDa protein was detected in the insoluble fraction of parasites lysate (Figure 2A, lane 3). No specific reaction was detected with pre-immune and anti-GST sera (Figure 2, lanes 5-6 and data not shown, respectively). To further confirm these results, we performed an immunoprecipitation assay using the parasite lysate with anti-BbTRAP rabbit immune serum. Consistently, the same three bands were detected by the blot analysis, indicating that these bands specifically correspond to the native BbTRAP2 (Figure 2B, lane 1). Interestingly, two bands with 76-, and 44-kDa were precipitated using the rabbit anti-rBbTRAP2 serum from the supernatant of the culture (Figure 2B, lane 3), indicating that the native BbTRAP was proteolytically processed and shed into the culture. Next, an IFAT was undertaken to determine the cellular localization of BbTRAP2 in *B. bovis* merozoites using the anti-rBbTRAP2 immune serum. The localization of BbTRAP2 was observed in the apical end of intracellular parasites by confocal laser microscopy (Figure 3A-B). Moreover, staining of the live parasites demonstrated specific fluorescence on the free merozoites (Figure 3C-D), indicating the translocation of this protein to the surface of the parasites. To ascertain our finding, we used anti-BbAMA1 serum as microneme marker and examined by confocal laser microscopy. The BbAMA-1 was detected in a pattern overlapped by BbTRAP2 at the apical end and surface of parasites (Figure 4A-C). These results may suggest that BbTRAP2 is a microneme protein located within the apical end of the parasites. The surface translocation of BbTRAP2 may allow its adhesive domains to directly interact with host cells. To further examine the ability of BbTRAP2 to bind to the host cells, bovine RBCs were incubated with a *B. bovis* lysate, and the precipitated proteins with the erythrocytes were identified by the specific serum. Strikingly, the Western blot analysis demonstrated two bands corresponding to 104- and 44-kDa proteins in the protein-bound erythrocyte lysate (Figure 5). Consistently, RBCs ghost membrane precipitated the same proteins (Figure 5), which may suggest that BbTRAP2 is functional in the merozoite stage and may be involved in the attachment to bovine RBCs. 

### Purified anti-rBbTRAP2 IgG inhibited the *in vitro* growth and invasion of *B. bovis*

**Figure 6:**
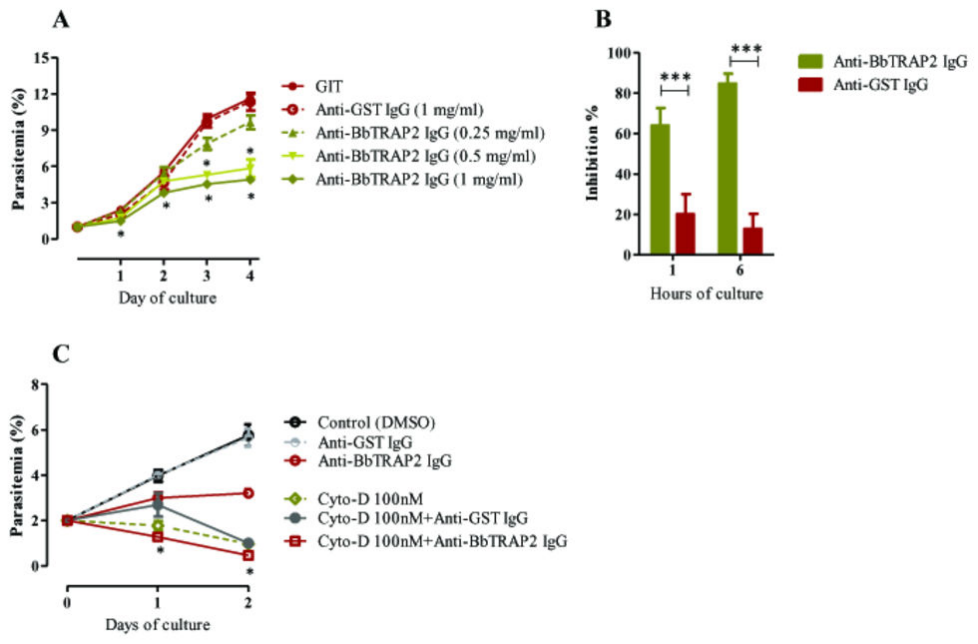
Effects of purified antibody to rBbTRAP2 on the growth and invasion of *B. bovis* *in* *vitro*. **A**, Growth inhibitory assay of *B. bovis* with different concentrations of IgGs. Parasitemias were determined daily for 4 days of culture. **B**, *B. bovis* invasion inhibition assay. Parasitemias were determined after 1 and 6 h of culture. **C**, Growth inhibitory assay of *B. bovis* in the presence of antibodies and Cytochalasin D. The means of parasitemia were statistically analyzed, and each asterisk indicates a significant difference (*P* < 0.05). Results represent two repeated experiments.

The inhibitory effects of anti-rBbTRAP2 purified IgG were evaluated on the *in vitro* growth of cultivated *B. bovis*. Of note, the addition of anti-rBbTRAP2 IgG inhibited the parasite growth in a concentration-dependent manner (Figure 6A). At 1 mg/ml of IgG, parasitemias were significantly inhibited (>40%) over the period of cultivation, while at 0.5 mg/ml, the significant inhibition was observed on days 3 and 4 post-culture (*P* < 0.05) as compared to those of the controls. In contrast, cultures containing anti-GST IgG as control antibody exhibited normal growth of parasitemias similar to those observed in control cultures without antibody. These results suggested that anti-rBbTRAP2 IgG could neutralize the parasites and restrict their *in vitro* growth. This finding raised the question of whether BbTRAP2 is involved in RBC invasion. To answer this question, free merozoites were pre-incubated with 1 mg/ml of anti-rBbTRAP2 IgG before adding to bovine RBCs. Thereafter, the parasitemias were determined at 1 and 6 h post-culture to cover the first life cycle of cultivated *B. bovis* [30]. Significant inhibition in the parasitemias was observed in the anti-rBbTRAP2-IgG-treated culture with maximum inhibition (approximately 80%) at 6 h post-culture (Figure 4B). Moreover, confocal laser microscopic observation of blood smears obtained 1 h post-culture demonstrated that the free merozoites were neutralized by anti-rBbTRAP2 IgG (data not shown). In contrast, no fluorescence was observed on the merozoite pre-incubated with the anti-GST serum (data not shown). These results suggested that the growth inhibition was probably due to the ability of anti-rBbTRAP2 IgG to neutralize free merozoites and disturb their invasion. Next, to examine the correlation between the inhibition by antibody and the parasite gliding motility, cultivated *B. bovis* was treated by Cytochalasin D either coupled antibody or without. Strikingly, culture with 25 or 100 nM Cytochalasin D and 1 mg/ml anti-BbTRAP2 IgG exhibited a marked reduction in parasitemia over two days of culture as compared to these in culture treated by the inhibitor or antibody alone (Figure 6C and Figure S3). These results may suggest that the inhibition by antibody is independent of the gliding of parasites. Furthermore, the potent inhibitory effect of anti-rBbTRAP2 IgG was compared to that of a set of several other antibodies prepared against recombinant BbMSA-2c, BbRAP-1CT and BbSBP-1. Antibodies to BbMSA-2c and BbRAP-1CT that were noted earlier to be inhibitive to the growth of parasites were used as positive controls [28,29]. The cultures with 1 mg/ml concentration of each antibody showed variant inhibitions on the growth of parasites. Strikingly, the antibody to rBbTRAP2 was the most inhibitive among the antisera used (Table 1). In particular, the inhibitions were statistically different on days 3 and 4 post-culture (*P* < 0.05). These data strongly suggested that BbTRAP2 might be a potential vaccine candidate against *B. bovis* infection. 

**Table 1:** Growth inhibitory effects of specific antibodies produced against recombinant proteins of the *B. bovis* erythrocytic stage.

**Antibody**	Growth inhibition (%) over the period of cultivation			
**1 mg/ml IgG**				
	Day 1	Day 2	Day 3	Day 4
**GST**	9.5±9.9	5.3±5.5	10.7±7.4	10.5±3.7
**BbTRAP2**	55.3±6.1^*^	48.1±5.6^*^	59.1±4.4^*^, ^**^	73.6±4^*^, ^**^
**BbRAP-1CT**	54.2±9.1^*^	45.9±8.2^*^	45.3±4.7^*^	60.9±5.4^*^
**BbMSA-2c**	19.2±17.9	17.7±8.2	26.1±8.9^*^	43.1±5^*^

^*^Asterisk indicates a significant difference (*P* < 0.05) in the rate of inhibition caused by test antibody as compared control antibody alone. ^**^ Two asterisks indicate a significant inhibition of anti-BbTRAP2 antibody as compared to other three antibodies used.

## Discussion

Considerable research efforts have been focused on vaccine development against *B. bovis* infection; however, live attenuated vaccines are still the most effective, and no alternative means have been marketed [4]. Immunization trials based on a non-living component, including parasite lysate and exoantigens derived from the supernatant of the culture, demonstrated potent protection in animals [31,32]. Likewise, immunization with immunodominant antigens of *B. bovis* parasites, such as rhoptry-associated protein (RAP-1), confers partial protection against virulent challenge infection [5]. Moreover, non-immunodominant proteins were also postulated to be appropriate candidates for eliciting protective immune responses against *Babesia* infection [8]. These reports suggest that a protective vaccine could be feasible using appropriate immunogenic antigens. Recently, annotation of genome sequences provided beneficial information for the identification of novel protective antigens needed for development of a recombinant subunit vaccine [8]. Proteins with a defined role in RBC invasion are of much interest as potential vaccine targets [9,11]. In the current study, a new member of the *B. bovis* BbTRAP family (P18) was identified and characterized as blood-stage vaccine candidate.

BbTRAP2 shares several conserved structural features of apicomplexan TRAPs because of the presence of vWFA and TSP1 domains at the N-terminus. These domains are identified in certain vertebrate molecules to function in cell–cell and cell–matrix interactions, blood coagulation, and innate immunity [12]. Therefore, the presence of these domains in BbTRAP2 as well as other protozoan TRAPs has suggested their potent role in interaction with a variety of vertebrate host and/or arthropod vector ligands during parasite invasion [14]. In order to functionally and biologically characterize BbTRAP2, the recombinant protein and its antiserum were produced. Contrary to the previously reported BbTRAP [18] that showed no/weak reaction to *B. bovis*-infected sera [21], BbTRAP2 had a strong reaction to the same samples, indicating its high immunogenicity. This might be explained based on the higher expression of the *BbTRAP2* gene in blood stage and characteristics of selected region sharing epitopes with other BbTRAPs. However, the better antigenicity of BbTRAP2 is in agreement with a related study that BgTRAP (BgP18) is a highly immunogenic protein in *B. gibsoni* infection [33]. Such a characteristic is important for the development of a subunit vaccine [5,8]. 

Western blot analyses using the parasite lysate and the culture supernatant revealed the proteolytic processing of BbTRAP2 into 104-, 76-, and 44-kDa proteins. An immunofluorescence assay on the localization studies demonstrated that BbTRAP2 was abundantly expressed in the merozoite stage with apical localization in the intracellular parasites and also with predominantly surface localization on the extracellular parasites. These findings differed from an earlier study noting that BbTRAP was translocated to the parasite surface and excreted into the supernatant of the culture without proteolytic processing [18]. However, the failure in detecting cleaved BbTRAP in that study is probably due to the antibody used to probe the lysate of parasites for targeting the short sequence of the protein. In the same regard, PfMTRAP was found to be expressed in middle-to-late asexual blood stages and to be localized initially in the micronemes and released onto the merozoite surface before RBC invasion [24,34]. The PfTRAP is deployed onto the parasite’s apical surface, where it is arranged in a cap- or ring-like pattern on the sporozoite, allowing its interaction with host receptor on the hepatocytes [14]. Likewise, a punctate pattern of micronemal proteins including AMA-1 and TRAP was observed on the surface of *Toxoplasma* tachyzoites and *Eimeria* sporozoites to mediate the parasite adhesion and the invasion into host cells [35,36]. The proteolytic cleavage of those micronemal proteins and their translocation to the surface of the parasites seem to be the central features of invasion by apicomplexan parasites [35-37]. After the cleavage of TRAPs by certain proteases, they rapidly redistribute toward the posterior at the site of attachment and hence the parasites initiate the actin–myosin-driven locomotion for their invasion machinery [15,35]. Rhomboids are serine proteases found in apicomplexan parasites and have been proposed to mediate the cleavage of TRAP homologues [15,16]. Interestingly, at least five homologues encoding rhomboids were found in the database of *B. bovis* T2Bo (http://www.genome.jp/kegg/kegg2.html), which may suggest their roles in RBC invasion. In support of this concept, serine protease inhibitors block the invasion of *B. divergens* into human RBCs [38]. Further study investigating the interaction between BbTRAP2 and *B. bovis* rhomboids is needed to understand the biological machinery of RBC invasion by the parasite. 

The localization of BbTRAP2 on the surface of merozoites may allow a direct interaction between its functional domains of vWFA and TSP1 and the host cells’ receptors. The RBC binding assay showed that bovine RBCs specifically precipitated two proteins (104- and 44-kDa), which correspond to native BbTRAP2 detected from the parasite lysate. The inability to precipitate the 76-kDa protein might be due to the absence of a specific ligand within this fragment. Further study is needed to identify the particular region of BbTRAP2 mediating the binding to the host RBCs. Consistently, Zhou et al. showed that the *B. gibsoni* TRAP bound intact canine RBCs in a bivalent cation-independent manner [19]. In a closely related parasite, *P. falciparum* merozoite and sporozoite TRAP is capable of binding human RBCs and hepatocytes, respectively, through the TSP1 domain [24,39]. Taken together, the newly identified BbTRAP2 seems to be functional in the merozoite stage and possibly mediates attachment of the parasites with bovine RBCs. Moreover, apicomplexan TRAPs are also known to play a central role in the gliding motility process that allows the trafficking and migration of the parasites into the host cells. These molecules serve as bridges to the host cells via the cytoplasmic tail, namely tryptophan residue, linked to the actomyosin motor of the parasites [10,12,14,17,40]. A recent study documented the process of gliding motility in *B. bovis* merozoites using time-lapse video microscopy [41]. This finding coupled with the presence of conserved tryptophan residue within the cytoplasmic tail of BbTRAP2 raises a question whether the inhibition by antibody is caused by perturbing the gliding motility of *B. bovis*. To answer this question, we have performed the growth inhibitory assay of the parasites in the presence of Cytochalasin D. Our results revealed that the inhibition by anti-BbTRAP2 serum is most probably independent of gliding motility. 

Furthermore, the potent inhibitory effect of the specific antibody to rBbTRAP2 was evaluated on the *in vitro* growth and invasion of the parasite. Consistent with its role in attachment to host cells, significant reductions in the growth and invasion of parasites were observed in a concentration-dependent manner. Interestingly, the anti-rBbTRAP2 immune serum was the most inhibitive to growth of the parasite as compared to those with a set of other antisera prepared against BbMSA-2c, BbRAP-1, and BbSBP-1. It is likely that free merozoites were neutralized by the anti-rBbTRAP2 serum and consequently lost their capability to invade the host RBCs. These results are in agreement with a previous study showing that a polyclonal rabbit antiserum against synthetic peptides derived from the TSP1 domain of BbTRAP1 inhibited RBC invasion [18]. In addition, the antibody to BgTRAP significantly suppressed the *in vivo* growth of *B. gibsoni* parasites in SCID mice [19]. Likewise, antibodies to PfTRAP significantly reduced the invasion of sporozoites into hepatocytes [14]. 

For the vaccine development, the ideal vaccine candidate against blood-stage *B. bovis* should target the molecule responsible for the induction of antibodies that prevent the invasion into the erythrocytes and cellular immune responses that mediate the clearance of the infected erythrocytes [8]. Since BbTRAP possesses such characteristics, we consider that this molecule can be a promising vaccine candidate and it is worthy in future to examine its protective effects against infection in cattle. 

In conclusion, a newly identified *B. bovis* thrombospondin-related anonymous protein is functional in the merozoite stage and is shed upon invasion to mediate the attachment to the host RBCs. The strong inhibitory effects of the antiserum on the growth and invasion of *B. bovis* suggest the potential of BbTRAP2 as a molecular vaccine target for *B. bovis* infection. Further study should evaluate the protective efficacy of BbTRAP2 immunization with appropriate adjuvant against challenge infection *in vivo*. 

## Supporting Information

Figure S1 **Graphic depiction of the predicted structure of BbTRAPs expressed in the merozoite stage.** **A**, Structure of BbTRAP1 (GenBank accession number XP_001609788.1), BbTRAP2 (GenBank accession number XP_001609812.1), BbTRAP3 (GenBank accession number XP_001609786.1), and BbTRAP4 (GenBank accession number XP_001609810.1). The BbTRAPs share a structure typical of apicomplexan TRAPs with the presence of vWFA, TSP1 domains, and a transmembrane domain followed by a cytoplasmic C-terminal tail. Only BbTRAP4 seems to lack the TSP1 domain. **B**, Multiple sequence alignment of the C-terminal regions of *BbTRAPs* deduced amino acid and other apicomplexan parasites TRAPs. Arrow indicates the conserved tryptophan residues. **C**, Transcriptional expression of *BbTRAPs* in the erythrocytic stage determined by RT-PCR and analyzed by Image J software.
(TIF)

Figure S2 **Multiple sequence alignment of the targeted BbTRAP2 with and other BbTRAPs.**
(TIF)

Figure S3 **Growth inhibitory assay of *B. bovis* in the presence of antibodies and Cytochalasin D.** The means of parasitemia were statistically analyzed, and each asterisk indicates a significant difference (*P* < 0.05).
(TIF)

Table S1 **Primer sequence for amplifying *BbTRAPs* in RT PCR.**
(DOC)
