# Supplementary figures and images for: Molecular Characterization of a New Babesia bovis Thrombospondin-Related Anonymous Protein (BbTRAP2)
Source: PLoS One. 2013 Dec 13;8(12):e83305. doi: 10.1371/journal.pone.0083305 (PMC3862764; doi:10.1371/journal.pone.0083305)

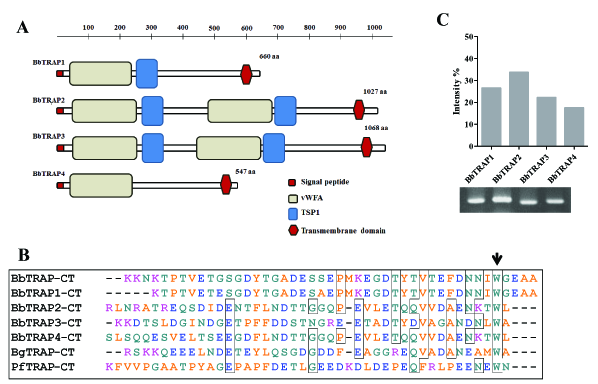

Supplement: Figure S1 — Graphic depiction of the predicted structure of BbTRAPs expressed in the merozoite stage. A, Structure of BbTRAP1 (GenBank accession number XP_001609788.1), BbTRAP2 (GenBank accession number XP_001609812.1), BbTRAP3 (GenBank accession number XP_001609786.1), and BbTRAP4 (GenBank accession number XP_001609810.1). The BbTRAPs share a structure typical of apicomplexan TRAPs with the presence of vWFA, TSP1 domains, and a transmembrane domain followed by a cytoplasmic C-terminal tail. Only BbTRAP4 seems to lack the TSP1 domain. B, Multiple sequence alignment of the C-terminal regions of BbTRAPs deduced amino acid and other apicomplexan parasites TRAPs. Arrow indicates the conserved tryptophan residues. C, Transcriptional expression of BbTRAPs in the erythrocytic stage determined by RT-PCR and analyzed by Image J software. (TIF) [file pone.0083305.s001.tif]

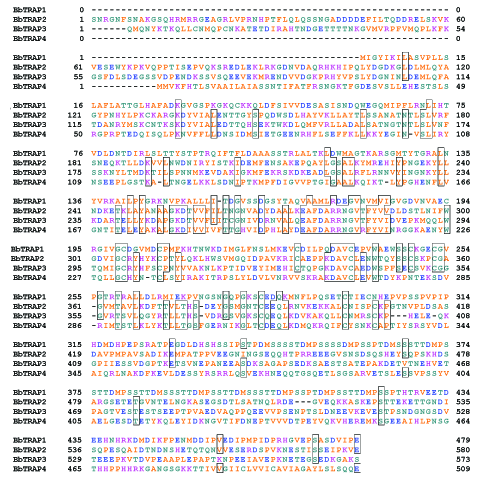

Supplement: Figure S2 — Multiple sequence alignment of the targeted BbTRAP2 with and other BbTRAPs. (TIF) [file pone.0083305.s002.tif]

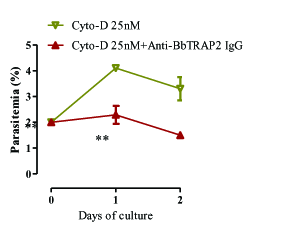

Supplement: Figure S3 — Growth inhibitory assay of B. bovis in the presence of antibodies and Cytochalasin D. The means of parasitemia were statistically analyzed, and each asterisk indicates a significant difference (P < 0.05). (TIF) [file pone.0083305.s003.tif]
