# Supplementary material for: Molecular Characterization of a New Babesia bovis Thrombospondin-Related Anonymous Protein (BbTRAP2)
Source: PLoS One. 2013 Dec 13;8(12):e83305. doi: 10.1371/journal.pone.0083305 (PMC3862764; doi:10.1371/journal.pone.0083305)
Supplement: Table S1 — Primer sequence for amplifying BbTRAPs in RT PCR. (DOC) [file pone.0083305.s004.doc]

**Table S1.** Primer sequence for amplifying *BbTRAPs* in RT PCR.

| **Gene** | **Primer** | **Product length** |
| --- | --- | --- |
| *BbTRAP1* | GTACCAGGCTCGCACTCACTA  AACGCCACATTCCCCTTTAC | 443 bp |
| *BbTRAP2* | AGAAGCCGGGCGTTTGGTG  AGTAGCGTTGGAGAAGATAGTGT | 460 bp |
| *BbTRAP3* | ATCCGCGCCCATACTAAT  GAGAGGGCATCAGCGAGAA | 410 bp |
| *BbTRAP4* | TTTCTGGAAGCGCCCGTGTAG  ACCGCCCGTAGTGTCGTTCAA | 428 bp |
